# *Wnt1* Role in the Development of the Habenula and the Fasciculus Retroflexus

**DOI:** 10.3389/fcell.2021.755729

**Published:** 2021-10-14

**Authors:** Verónica Company, Ana Moreno-Cerdá, Abraham Andreu-Cervera, Raquel Murcia-Ramón, Francisca Almagro-García, Diego Echevarría, Salvador Martínez, Eduardo Puelles

**Affiliations:** Instituto de Neurociencias de Alicante, Universidad Miguel Hernández-CSIC, Sant Joan d’Alacant, Spain

**Keywords:** *Wnt1*, habenula, fasciculus retroflexus, proliferation, differentiation

## Abstract

*Wnt1* is one of the morphogenes that controls the specification and differentiation of neuronal populations in the developing central nervous system. The habenula is a diencephalic neuronal complex located in the most dorsal aspect of the thalamic prosomere. This diencephalic neuronal population is involved in the limbic system and its malfunction is related with several psychiatric disorders. Our aim is to elucidate the *Wnt1* role in the habenula and its main efferent tract, the fasciculus retroflexus, development. In order to achieve these objectives, we analyzed these structures development in a *Wnt1* lack of function mouse model. The habenula was generated in our model, but it presented an enlarged volume. This alteration was due to an increment in habenular neuroblasts proliferation rate. The fasciculus retroflexus also presented a wider and disorganized distribution and a disturbed final trajectory toward its target. The mid-hindbrain territories that the tract must cross were miss-differentiated in our model. The specification of the habenula is *Wnt1* independent. Nevertheless, it controls its precursors proliferation rate. *Wnt1* expressed in the isthmic organizer is vital to induce the midbrain and rostral hindbrain territories. The alteration of these areas is responsible for the fasciculus retroflexus axons misroute.

## Introduction

The habenula (Hb) is a phylogenetically conserved bilateral nucleus (Aizawa et al., 2011; Schmidt and Pasterkamp, 2017; Zahm and Root, 2017). This structure is at the most dorsal part of the diencephalic thalamic prosomere (p2; Puelles, 2019), and together with the pineal gland and the stria medullaris (sm) comprises the epithalamus (Hikosaka, 2010; Schmidt and Pasterkamp, 2017; Zahm and Root, 2017). The Hb is divided into the medial habenula (mHb) and the lateral habenula (lHb) that differ among them in their connectivity, neurochemical characteristics, and related functions (Andres et al., 1999; Schmidt and Pasterkamp, 2017; Zahm and Root, 2017; Roman et al., 2020). The lHb is the larger one in surface but the lower in neuronal density (Hashikawa et al., 2020; Wallace et al., 2020). This original subdivision has been further subdivided into different subpopulations defined by transcriptomics. Thus, the lHb is divided into nine differential subpopulations and the mHb into six subpopulations plus an intermediate subpopulation named HbX (Wagner et al., 2016). These subdivisions have been simplified but corroborated by single cell RNAseq experiments (Pandey et al., 2018; Hashikawa et al., 2020; Wallace et al., 2020).

The vertebrate Hb is one of the components of the dorsal diencephalic conduction system, a highly conserved limbic pathway that links the forebrain with the monoaminergic system (Sutherland, 1982; Zahm and Root, 2017; Fakhoury, 2018; Roman et al., 2020). This system originates in the forebrain, projects through the sm to the Hb complex that projects to the interpeduncular nucleus (Ip) in the hindbrain and monoaminergic nuclei in the midbrain, mainly through the fasciculus retroflexus (fr), the main Hb efference (Zahm and Root, 2017). This tract develops following a complex trajectory (Moreno-Bravo et al., 2016). Due to its projections toward these mesencephalic monoaminergic nuclei, the Hb has a role in their functional regulation (Schmidt and Pasterkamp, 2017; Zahm and Root, 2017). This fact implies the Hb in behaviors such as learning, social behavior, value-based decision making or avoidance of negative stimuli (Okamoto et al., 2012, 2021; Amo et al., 2014; Koppensteiner et al., 2016; Schmidt and Pasterkamp, 2017; Loonen and Ivanova, 2019; Cherng et al., 2020; Hu et al., 2020; Nakajo et al., 2020). It is also involved in mechanisms related to pain modulation, fear and anxiety, helpless behavior, mood disorder and drug addiction (Agetsuma et al., 2010; Lee et al., 2010; Jesuthasan, 2012; Okamoto et al., 2012; Mathuru and Jesuthasan, 2013; Batalla et al., 2017; Mathuru, 2018; Roman et al., 2020). Finally, it is also implicated in some psychiatric disorders including major depression or schizophrenia, as well as some neurological disorders like Parkinson’s disease (Hikosaka, 2010; Nakajima et al., 2013; Benarroch, 2015; Schmidt and Pasterkamp, 2017; Hu et al., 2020).

The Hb receives inputs from limbic forebrain areas as the septum, basal ganglia and hypothalamus among others through the sm. Once these inputs are integrated in the Hb, efferent fibers project through the fr. This tract consists of two regions, a core of mHb axons and a shell of fibers from the lHb (Roman et al., 2020). The core innervates the Ip nucleus after crossing the floor plate several times (Ramón y Cajal, 1909; Contestabile and Flumerfelt, 1981; Klemm, 2004). Finally, lHb axons innervate monoaminergic areas, the rostromedial tegmental nucleus, the substantia nigra pars compacta (SNc), and the ventral tegmental area (VTA) in the midbrain involved in the release of dopamine (Jhou et al., 2009). Meanwhile, axons from the Ip nucleus project to the median and dorsal raphe nuclei, involved in the release of serotonin. This tract also has ascending projections from SNc toward the lHb (Hikosaka, 2010; Schmidt et al., 2014; Fakhoury, 2018). These data demonstrate that the Hb is an information relay station between the forebrain and the midbrain and hindbrain (Bianco and Wilson, 2009; Schmidt and Pasterkamp, 2017; Fore et al., 2018; Grillner et al., 2018; Loonen and Ivanova, 2019) and it is involved in the modulation of catecholaminergic system (Hikosaka et al., 2008; Hikosaka, 2010).

Complex molecular programs control the morphogenesis and the wiring of the developing Hb (Schmidt and Pasterkamp, 2017). The Hb anlage derives in vertebrates from the alar plate of p2 (Martinez-Ferre and Martinez, 2012; Beretta et al., 2013), a domain defined by the expression of specific transcription factors. Three families of morphogenes confluence in this Hb primordium for its specification, differentiation and proliferation: Fibroblast growth factors (Fgfs), bone morphogenetic proteins (Bmps), and wingless-int factors (Wnts; Masai et al., 1997; Barth et al., 1999; Kazanskaya et al., 2000; Regan et al., 2009).

*Wnt* family is a group of genes that are involved in embryonic development, especially in controlling the embryonic pattern (Ciani and Salinas, 2005; Bengoa-Vergniory and Kypta, 2015; Brafman and Willert, 2017). This family is also involved in cell differentiation, polarization, migration during development and programmed cell death (Ciani and Salinas, 2005; Brafman and Willert, 2017; Taciak et al., 2018).

*Wnt* signaling acts together with other signal molecules. It is required for the induction of the midbrain-hindbrain boundary, which is an important organizing center (Ciani and Salinas, 2005; Brafman and Willert, 2017). In this case, it acts together with *Fgf8* in a regulatory network (Brafman and Willert, 2017). *Wnt1* is expressed in the dorsal part, and it is necessary for the maintenance of *Fgf8* expression. This molecule is essential for the induction of the midbrain and the cerebellum (Ciani and Salinas, 2005). Its direct role in the differentiation program of the mid-diencephalic dopaminergic neurons has been demonstrated (Prakash et al., 2006). Besides, it has been observed that *Wnt1* is responsible for the regionalization of the diencephalon being also expressed along this region until the p2/p3 boundary. In fact, in the absence of *Wnt1*, an altered diencephalic structure is observed because it is needed for a proper DV patterning (Navarro-Garberi et al., 2016). Recent studies report evidence for the requirement of the Wnt pathway in the development of the habenular complex (Schmidt and Pasterkamp, 2017; Roman et al., 2020). In Zebrafish, it has been demonstrated that *Wnt1* function is required for the correct specification of the dorsal Hb medial (equivalent to a subnucleus of the murine mHb; Beretta et al., 2013; Hüsken and Carl, 2013; Guglielmi et al., 2020). Moreover, the alteration of *wls*, a gene that codifies a protein necessary for WNT secretion, results in a dHb reduction and the absence of vHb (Kuan et al., 2015).

All this information prompted us to hypothesize that *Wnt1* plays a key role in the specification and differentiation of the habenular complex as well as in the formation of the fr, its main efference. In order to demonstrate this hypothesis, we studied the role of *Wnt1* in the habenular and fasciculus retroflexus embryonic development using a mouse model null for *Wnt1* compared to a wild type (wt).

## Materials and Methods

### Mouse Strains

For staging, the day when the vaginal plug was detected was considered as embryonic day 0.5 (E0.5). All mouse manipulation and experimental procedures were performed according to the directives of the Spanish and European Union governments and the protocols were approved by the Universidad Miguel Hernández OIR Committee (2016/VSC/PEA/00190). The *Wnt1^–/–^* transgenic mouse line generation and genotype were previously described in McMahon et al. (1992).

### Bromodeoxyuridine Injections

Pregnant mice were intraperitoneally injected 5 μl/gr of body weight with Bromodeoxyuridine (BrdU; 5 mg/ml) at the desired gestational stage (E11.5-E13.5) and sacrificed 2 h later. Embryos were fixed overnight in 4% Paraformaldehide (PFA) and washed in Phosphate Buffered Saline (PBS).

### Axonal Tracing

Brains were fixed 1 h in 4% PFA and embedded in 4% agarose in PBS. Afterward, brains were sectioned in coronal plane with a vibratome until reaching the habenular nucleus. At this point, the fluorescent crystals were placed in both habenulae: DiI (1,1′-dioctadecil 3,3,3′,3′-tetra-metilindocarbo-cianina perchlorate; Molecular Probes) on one side and DiD (1′-dioctadecyl-3,3,3′,3′- tetramethylindodicarbocyanine, 4-chlorobenzenesulfonate salt; Molecular Probes) on the contralateral side. Samples were left at 37°C in 4% PFA until crystal had diffused along the retroflexus tract. Labeled brains were sectioned in the vibratome and IHC processed without detergent to avoid the signal loss.

### Immunohistochemistry and *In situ* Hybridization

Mouse embryo brains were fixed overnight in PFA in PBS. Samples were agarose-embedded and sectioned at 100 μm in coronal or sagittal planes by vibratome or wax embedded and sectioned at 10 μm by microtome.

For IHC, it was performed as previously described (Murcia-Ramón et al., 2020). The primary antibodies used were: αTH (1:1,000; 208020234/Inst. J. BOY), αNTN-1 (1:500; MAB1109/RD Systems), αDCC (1:100; sc-6535/Santa Cruz), αCNTN2 (1:500; AF4439/RD Systems), αNFEM (1:500; AB1987/Chemicon), αBrdU (1:200; M0744/Dako), αCALB (1:1,000; CB-38/Swant), αROBO3 (1:300; AF3076/RD systems), αSOX2 (1:500; ab97959/Abcam) and NFEM (1:1,000; Ab7794/Abcam).

For ISH, embryonic brains were washed three times for 20 min with detergent mix (1% IGEPAL, 1% SDS, 0.5% Deoxycholate, 50 mM Tris pH 8, 1 mM EDTA, 150 mM NaCl). Afterward, brains were post fixed in 4% PFA and rinsed in PBS. The next step was the pre-hybridization with hybridization buffer (50% Deionized Formamide, 5x Salt sodium citrate (SSC) pH 5.3, 50 mg/ml Heparin, 0.1% Tween 20). Hybridization chamber was prepared with the pre-hybridization mix (50% formamide, 5x SSC, 50 μg/ml heparin, 50 μg/ml tRNA, 50 μg/ml ssDNA, 0.1% Tween-20) for 1 h at 65°C. Then, the Digoxigenin-labeled RNA probe (*Fgf8*, Addgene plasmid #22090) was denaturalized at 80°C and the tissue was incubated in hybridization buffer with the probe overnight at 65°C. Samples were washed 4 times for 30 min at 65°C with 2x SSC pH 5.3, 50% formamide, 1% SDS. Three additional washes of 5 min and one of 30 min were carried out with 1x MABT (MAB solution (500 mM Maleic acid, 750 mM NaCl, 0.95M NaOH pH 7.5) and 0.1% Tween-20). Next step was to incubate embryos with 2% RBR (Roche Blocking Reagent) and MABT for 1 h. Then, brains were blocked with 2% RBR, and 20% Sheep Serum in MABT for 1 h. Then, samples were incubated in a wet chamber overnight at 4°C with an alkaline phosphatase-coupled anti-digoxigenin antibody (11207733910/Roche) prepared in the blocking solution at 1:2,000. The following day, samples were extensively washed with MABT. Then, before the color reaction, samples were washed with NTMT (100 mM NaCl, 100 mM Tris-HCl pH 9.5, 50 mM MgCl_2_, 1% Tween20). The NBT/BCIP (Boehringer, Mannheim) was used as a chromogenic substrate to detect the probes. The alkaline phosphatase reacts with this substrate producing a solid precipitate.

### iDISCO

E18.5 fixed brains were boiled in 0.01M sodium citrate at 80°C for 8 min and dehydrated with 90 min methanol washes at increasing concentration (20, 40, 60, 80, 100%). Brains were left overnight in 100%methanol at room temperature (RT). Next day, brains were incubated overnight at 4°C in a solution consisting of 1/3 100% methanol and 2/3 DCM (dichloromethane). The following day, brains were washed twice with methanol 100% for 1 h. At this point, brains were incubated in a solution of 5% hydrogen peroxide prepared in 100% methanol overnight at 4°C. The next day, brains were rehydrated in descending methanol concentrations. After that, brains were washed twice for 1 h at RT with PTx2 (10x PBS, 2% TritonX-100) and were incubated overnight at 37°C with permeabilization solution (0.3 M glycine, 20% DMSO in PTx.2). After this time, brains were incubated overnight with blocking solution (6% donkey serum, 10% DMSO in PTx.2) at 37°C. Then, samples were incubated for 10 days at 37°C with the primary antibody solution: αTH (1:1,000; N° 268020234/Institute Jacques Boy Cat), αROBO3 (1:300; AF3076/RD systems). Brains were extensively washed with PTwH (0.2% Tween 20, 0.01% heparin in 1x PBS). Afterward, samples were incubated overnight at 37°C with the secondary antibody solution prepared at 1:500 (5% DMSO and 3% Donkey serum in PTwH). This solution was previously filtered with a 0.2 μm filter. The following day they were washed with PTwH and dehydrated with methanol. Once dehydrated, brains were incubated with a solution of 2/3 DCM and 1/3 100% methanol. Then, samples were immersed in DCM twice for 30 min and were stored in BDE (Benzyl ether) until being processed. In all the steps, the brains are kept in movement to improve the processes.

### Cresyl Violet Staining

Sections were immersed in Cresyl Violet for 3–4 min and were submerged twice in distilled water for 2–3 min. Then, samples were washed with ethanol at increasing concentration (70, 96, 100, and 100%). Finally, samples were immersed in xylol twice and covered with Eukitt Clasic Mounting Medium.

### Image Acquisition, Image Processing, and Data Analysis

Fluorescent images were acquired with confocal microscope Leica SPE II whereas bright field images were acquired with a camera (Leica DFC500) associated with a stereomicroscope (Leica MZ16FA). 3D imaging was performed with a light-sheet fluorescence microscope (Ultramicroscope II, LaVision BioTec) and processed with Imaris software X64 9.3.0. Figures were composed with Adobe System software (CS6).

The proliferation assay was quantified by the area occupied by the red fluorophore using the measure tool of the Fiji software (ImageJ 1.53C). To do this, the image was binarized and the percentage of area occupied by white points was measured. The habenular volumes were measured with the surface tool of the Imaris software.

Statistical analysis was performed with unpaired *t-*test using GraphPad Prism 8.4.3 software.

## Results

### *Wnt1^–/–^* Habenular General Phenotype

**FIGURE 1:**
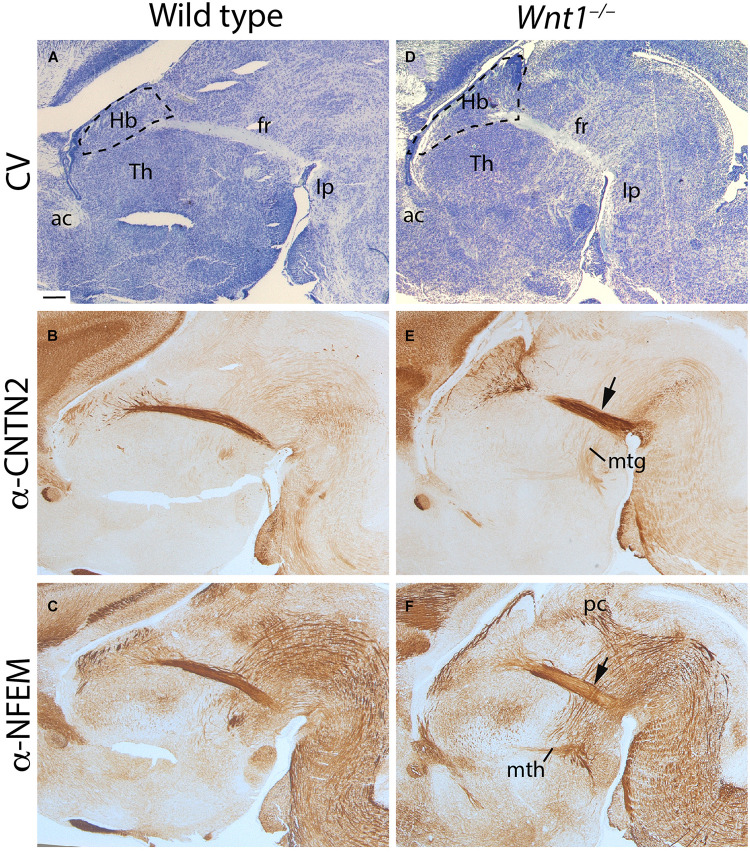
*Wnt1^–/–^* habenular general phenotype. E18.5 wt **(A–C)** and E18.5 Wnt1^–/–^ brain sagittal sections **(D–F)**. **(A,D)** Are stained with cresyl violet, **(B,E)** are labeled for CNTN2 (as mHb marker), and **(C,F)** for NFEM (as lHb marker). The tract was thicker (arrows in **E,F**), and the Hb was larger in the mutant embryo. Five samples of each genotype have been analyzed. fr, fasciculus retroflexus; Hb, habenula; mtg, mamillotegmental tract; mth, mamillothalamic tract; Th, thalamus; ac, anterior commissure; Ip, interpeduncular nucleus; pc, posterior commissure; CV, cresyl violet. Scale bar: 200 μm.

In order to have a first overview of the possible phenotype displayed by our mouse transgenic model, we analyzed E18.5 *Wnt1^–/–^* and wt brains in sagittal sections. In the wt, by cresyl violet staining, we observed the general histology of the brain. The Hb was located in the most dorsal aspect of the thalamic prosomere (Figure 1A). From its ventral tip, we detected the fr extending toward the diencephalic tegmentum before bending caudally toward its main hindbrain target, the Ip nuclei (Figure 1A; see Figure 1 in Moreno-Bravo et al., 2016).

With the aim to discern between the mHb and lHb axons, we studied the distribution of specific markers for each axonal projection. CNTN2 protein is specifically located in mHb axons (Figure 1B) and NFEM protein in lHb axons (Figure 1C). Note that NFEM distribution is slightly wider than CNTN2, this is due to the fact that mHb axons occupied the core of the fascicle meanwhile the lHb axons are located in the shell (compare Figures 1B,C). In *Wnt1^–/–^* brain, the Hb territory appeared wider in its anteroposterior axis and the thalamic territory displayed a clear size reduction (Figure 1D). The fr showed a slightly abnormal appearance (Figure 1D). Core and shell fr specific markers allowed us to distinguish a wider distribution of the mHb axons (arrow in Figure 1E) as well as in the case of the lHb axons (arrow in Figure 1F). Other diencephalic tracts such as the posterior commissure and the mammillothalamic and mammillotegmental tracts did not display any apparent alteration (Figures 1E,F).

### Habenular Volumes and Subnuclei Organization Analysis in *Wnt1^–/–^*

**FIGURE 2:**
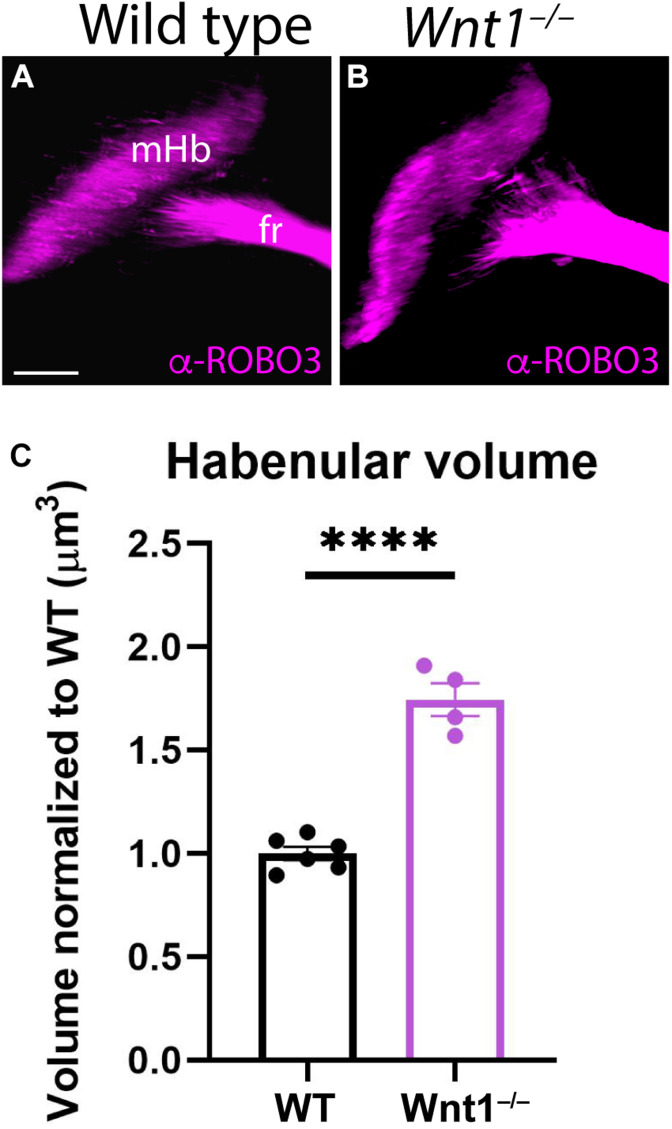
Medial habenular volumes analysis in *Wnt1^–/–^*. Lateral views of E18.5 wt **(A)** and *Wnt1*^–/–^ **(B)** brains analyzing ROBO3 distribution by iDISCO. We can observe the mHB and the initial fr portion. **(C)** Significance among wt (*n* = 6) and *Wnt1^–/–^* mHb volumes (*n* = 4) was analyzed by an unpaired *t*-test. Significant differences were found (^*⁣*⁣**^< 0.0001), being the mutant mHb volume larger than the wt. fr, fasciculus retroflexus; mHb, medial habenula. Scale bar: 200 μm.

To confirm our first results, we analyzed ROBO3 distribution (mHb marker) by iDISCO in E18.5 brains with the aim to calculate the mHb volume in mutants compared to wt embryos. In a lateral view of the scanned brain, we were able to observe the full extension of the mHb as well as the initial fr portion (Figure 2A). In the mutant brain, the mHb displayed an abnormal and enlarged shape (Figure 2B). Also, the initial fr portion displayed a wider appearance (Figure 2B). The iDISCO scanned brains allowed us to quantitatively measure the volume occupied by both mHb. The *Wnt1^–/–^* Hb volume was larger than the wt, being the difference among them statistically significant (Figure 2C; Unpaired *t*-test, *p* < 0.0001).

**FIGURE 3:**
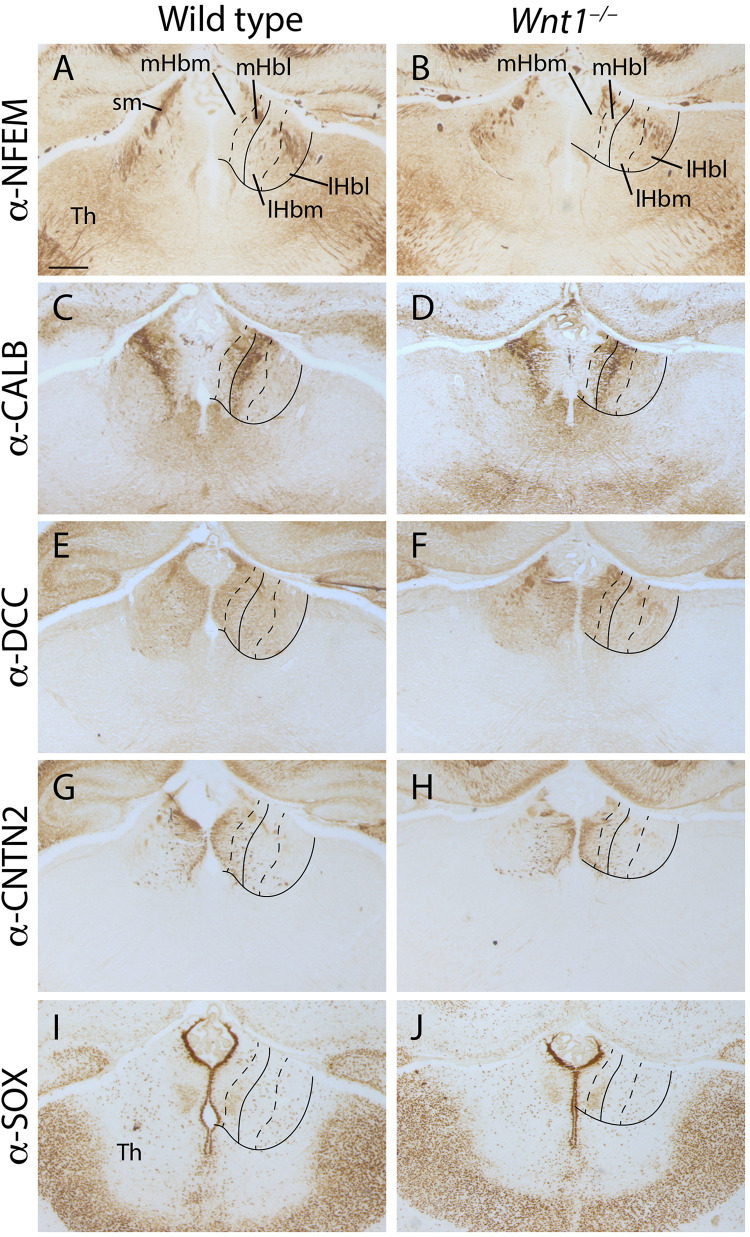
Analysis of the habenular subnuclei organization. E18.5 wt **(A,C,E,G,I)** and E18.5 *Wnt1^–/–^* **(B,D,F,H,J)** brain coronal sections. **(A,B)** labeled by immunohistochemistry against NFEM as lHb marker. **(C,D)** labeled against CALB, **(E,F)** DCC, **(G,H)** CNTN2 and **(I,J)** SOX1 as mHb markers. Four samples of each genotype have been analyzed. Th, thalamus; sm, stria medullaris; mHbm, medial division of the medial habenula; mHbl, lateral division of the medial habenula; lHbm, medial division of the lateral habenula; lHbl, lateral division of the lateral habenula. Scale bar: 200 μm.

Once we demonstrated the enlargement of the habenular territory, we aimed to analyze the main subdivisions distribution in the *Wnt1^–/–^* Hb compared to the wt. We grouped the different subnuclei in four divisions of the habenular complex. The mHb was formed by a medial division (mHbm) and a lateral division (mHbl) and the lHb was subdivided as well in a medial division (lHbm) and a lateral division (lHbl). Then, we analyzed several markers for the different territories. The NFEM protein distribution strongly labeled the sm in both samples (Figures 3A,B). It was absent from the mHb divisions meanwhile in the lHb divisions was weakly present, no obvious difference was found between the mutant and the wt (Figures 3A,B). CALB presented a complex pattern, it was strongly located in the medial aspect of the lHbm. Meanwhile in the mHbl presented a gradient distribution pattern from dorsal to ventral. Again, we did not find any obvious alterations between mutant and wt (Figures 3C,D). DCC, marker of the mHb, was homogeneously found in the mHb divisions and a weaker distribution in the lHb divisions. Again, no differences were found between samples (Figures 3E,F). CNTN2 protein, a mHb marker, strongly labeled the mHbm but also presented a scattered distribution in the mHbl. In the lHb divisions we observed some mHb axons fasciculating the initial fr. Once again, no significant difference was observed (Figures 3G,H). Finally, we analyzed SOX2 distribution pattern which specifically labeled the periventricular layer of the mHbm, and it specifically labeled in the ventrolateral subnuclei in the mHbl division. Once again, this labeling was observed in both samples (Figures 3I,J).

Summarizing, no defects were observed in the cellular organization of both mHb and lHb. Nevertheless, it must be pointed out that the mutant Hb displayed a general abnormal flattened shape when compared with the wt Hb.

### Proliferation Analysis of the Habenular Complex

With the aim to unveil the reason for the abnormal growth of the habenular territory, we did proliferation assays analysis. From E11.5 to E13.5 (time window for the habenula differentiation; Funato et al., 2000; Belle et al., 2014), we collected embryos 2 h after a BrdU pulse was injected intraperitoneally to the pregnant females on the given day. Thereafter, we compared the proliferation between wt and mutant embryos.

**FIGURE 4:**
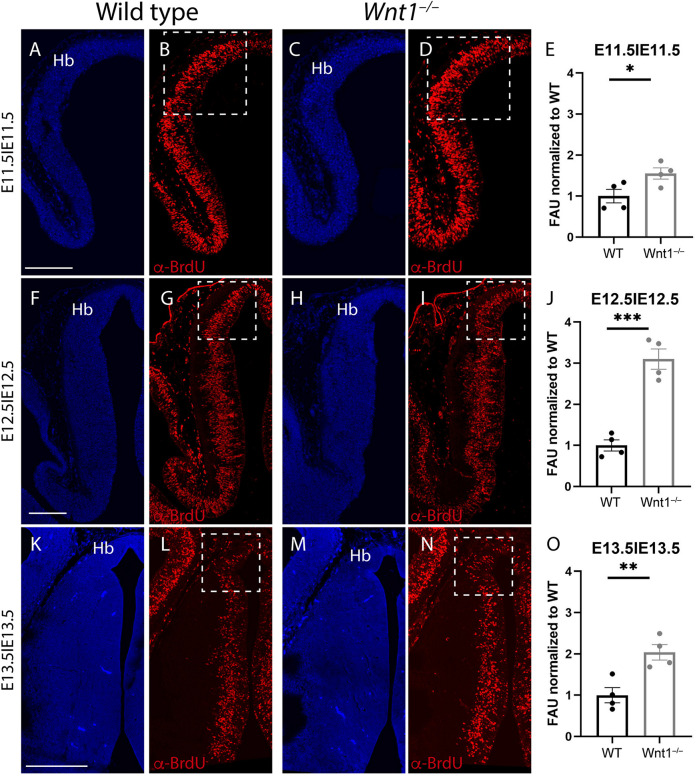
Proliferation analysis. Brain coronal sections at embryonic stages E11.5 **(A–D)**, E12.5 **(F–I),** and E13.5 **(K–N)**. Pregnant mice were injected with BrdU on the designated day. The embryos were collected 2 h later. Immunofluorescence against BrdU **(B,D,G,I,L,N)** showed more proliferation in the Wnt1^–/–^ brains than in the wt brains **(E,J,O)**. Significance among wt and Wnt1^–/–^ proliferation was analyzed by an unpaired *t*-test. Significant differences were found (**E** ^∗^< 0.05; **J** ^∗∗∗^< 0.001; **O** ^∗∗^< 0.01), being proliferation in the mutant higher than in the wt. Four samples of each genotype have been analyzed. Hb, habenula. Scale bar: 200 μm.

At E11.5, when we compared the wt (Figures 4A,B) with the *Wnt1^–/–^* (Figures 4C,D), the proliferation was slightly higher in the mutant (Figure 4E). However, at E12.5, the proliferation in the wt (Figures 4F,G) in contrast to the *Wnt1^–/–^* (Figures 4H,I) displayed the strongest difference (Figure 4J). Finally, at E13.5 the correlation between the control (Figures 4K,L) and the mutant (Figures 4M,N) also showed a significant variation (Figure 4O). Summarizing, the *Wnt1^–/–^* mice displayed a higher proliferation rate in the habenular territory in all the analyzed periods showing the highest difference at E12.5.

### Altered fr Trajectory in *Wnt1^–/–^*

**FIGURE 5:**
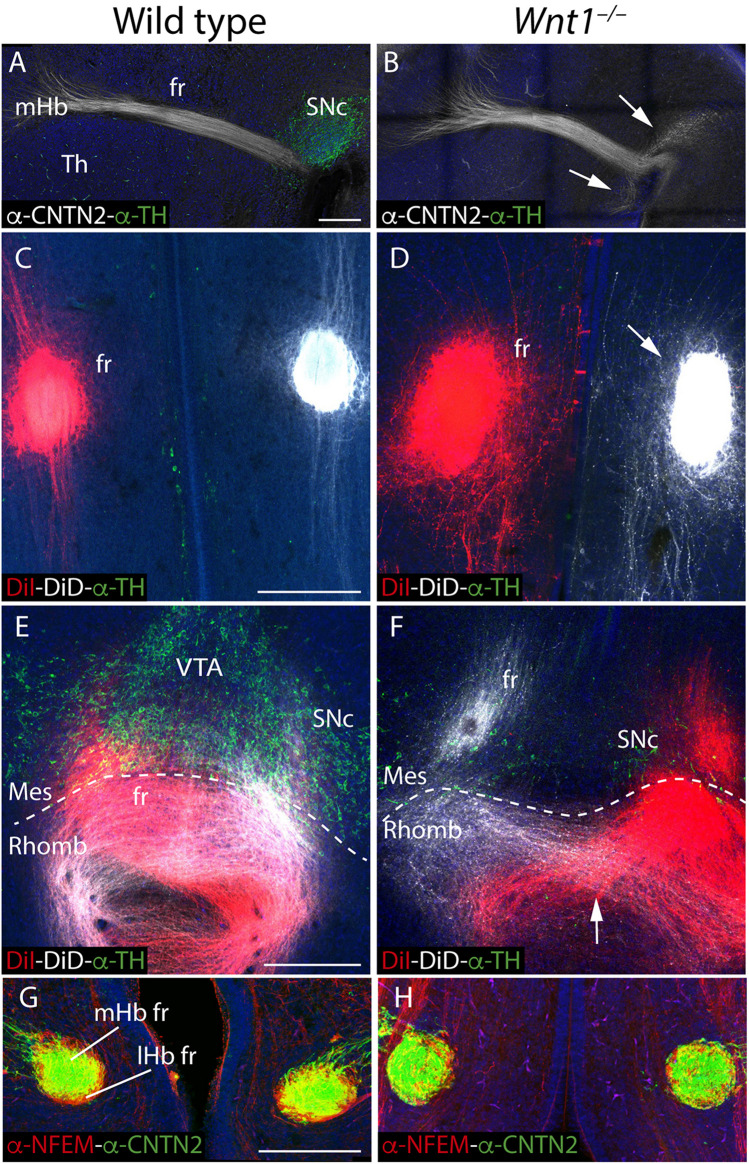
Study of the fr phenotype in *Wnt1^–/–^* mutant. E15.5 wt **(A)** and *Wnt1^–/–^* brain sagittal sections **(B)** stained against CNTN2 (mHb marker; white) and TH (green). DiI (right fr; red) and DiD (left fr; white) labeling and TH immunofluorescence (green) in coronal sections of an E15.5 wt **(C,E)** and *Wnt1^–/–^* mutant brain **(D,F)** at two different levels. Coronal sections of an E15.5 wt **(G)** and *Wnt1^–/–^* mutant brain **(H)**, stained with NFEM (as lHb marker; red) and CNTN2 (as mHb marker; green). The arrow in **(B)** indicates the aberrant axonal navigation. The arrow in **(D)** indicates the abnormal fr shape. The arrow in **(F)** indicates the abnormal cross of the floor plate. The dashed line labels the mid-hindbrain boundary. Four samples of each genotype have been analyzed. fr, fasciculus retroflexus; lHb lateral habenula; lHb fr, lateral habenular axons of the fasciculus retroflexus; Mes, mesencephalon; mHb, medial habenula; mHb fr, medial habenular axons of the fasciculus retroflexus; Rhomb, rhombencephalon; SNc, substantia nigra pars compacta Th, thalamus; VTA, Ventral tegmental area. Scale bar: 200 μm.

Having described the Hb phenotype, and the affected mechanism that underlined it, we focus our attention on the fr trajectory, main efferent tract of the Hb complex. First, we compared in sagittal sections the mHb fr trajectory (by CNTN2 labeling) with the SNc localization (by TH labeling) at E15.5. In the wt, the mHb reached the basal thalamic territory in close contact with the SNc (Figure 5A). However, in the *Wnt1^–/–^*, the SNc was strongly reduced and a thicker mHb fr mainly followed the normal trajectory (Figure 5B). Nevertheless, a bunch of fibers adopted abnormal directions (arrows in Figure 5B). In order to study the fasciculation and final trajectory of the full tract (mHb and lHb), we placed lipophilic dyes on E15.5 normal and mutant Hbs (DiI on the right Hb and DiD on the left Hb). In a diencephalic frontal section, we observed in the wt two compact fr, each one labeled with the specific dye (Figure 5C). In the *Wnt1^–/–^*, the fascicles occupied a broader domain and a slight defasciculation was detected (arrow in Figure 5D). It is known that in the last caudal portion of the fr, the fibers crossed the mes-rombencephalic boundary and the mHb axons criss-crossed the floor plate to innervate its target, the IP nucleus. Thus, in the wt, once the fibers crossed the dopaminergic territory, we nicely observed the characteristic eight shaped pattern of the mHb axons innervating the Ip nucleus (Figure 5E). Meanwhile, we confirmed in the *Wnt1^–/–^* mice that the dopaminergic neurons showed a dramatic reduction and an abnormal trajectory of the Hb fibers (Figure 5F). The Hb axons were able to go through the mid-hindbrain boundary, but once located in the rhombencephalon not all of them were able to cross the midline but only just one time (arrow in Figure 5F). Finally, we studied the distribution of mHb (labeled by CNTN2) and lHb axons (labeled by NFEM) in the fr. In the wt, the mHb axons occupied the core of the fascicle, whereas the lHb are distributed in the sheath (Figure 5G). In the *Wnt1^–/–^*, the mHb axons were still located in the core of the fascicle but the lHb axons presented a less compacted distribution and we found some intermingled fibers (Figure 5H).

**FIGURE 6:**
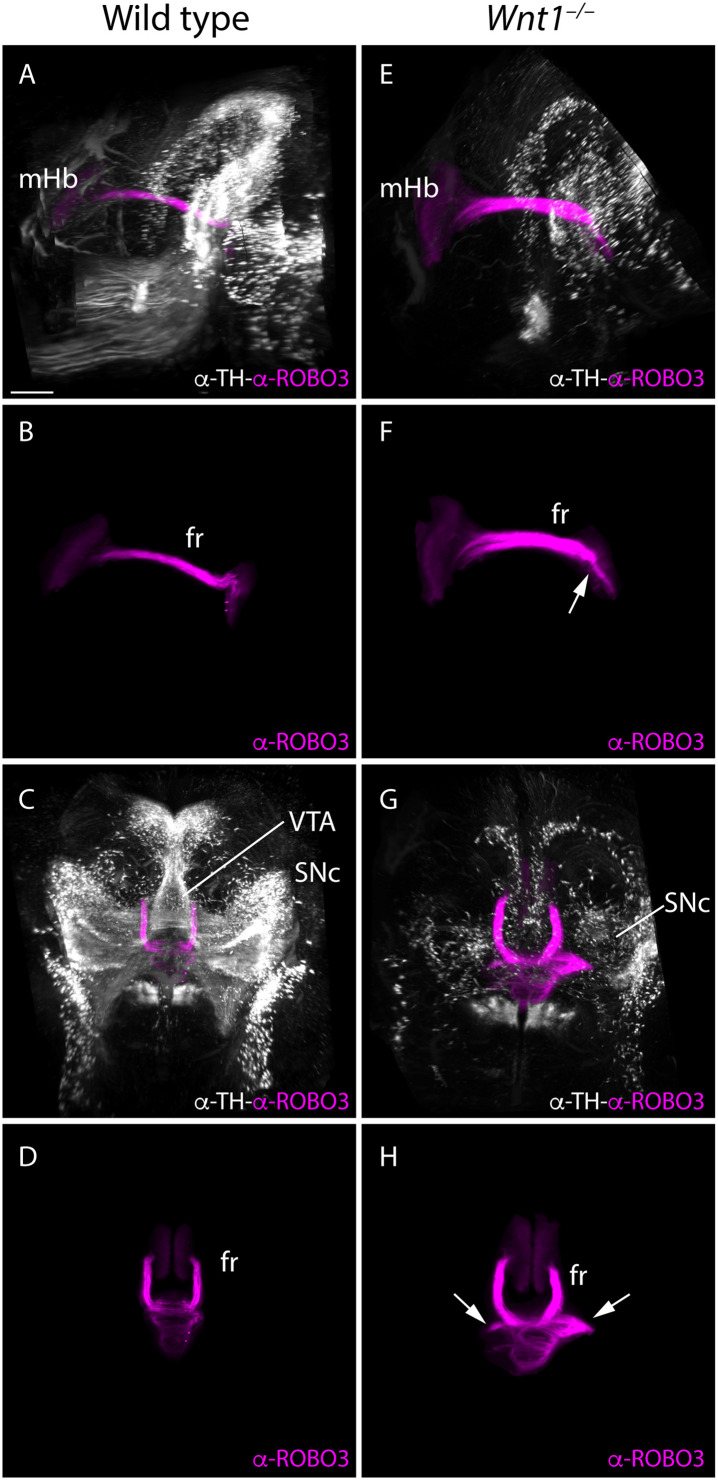
3D study of the fr and SNc phenotype in *Wnt1^–/–^.* Lateral **(A,B,E,F)** and frontal **(C,G,D,H)** view of E18.5 wt **(A–D)** and *Wnt1^–/–^* **(E–H)** brains labeled against TH (white) as SNc marker and ROBO3 (magenta) as mHb marker with iDISCO protocol. These 3D views allowed us to follow the trajectory of the mHb fr tract and its relation with the dopaminergic populations by TH. In the mutant **(G,H)**, we clearly observed the tract trajectory altered (arrows) and a drastic reduction of dopaminergic populations in the mid-diencephalic territory **(E,G)**. Six samples of each genotype have been analyzed. fr, fasciculus retroflexus; mHb, medial habenula; SNc, substantia nigra pars compacta; VTA, ventral tegmental area. Scale bar: 400 μm

Taking the technical advantage of observing the specimen in a 3D way we analyzed the full trajectory of the fr with iDISCO protocol. We processed E18.5 mutant and wt brains for whole mount immunohistochemistry with ROBO3 for mHb and its projections and TH for the dopaminergic populations. In the wt, in a lateral 3D vision we observed the complete trajectory, first in its dorso-ventral path and in its final rostro-caudal portion and how it is strongly related with the mes-diencephalic dopaminergic neurons (Figures 6A,B and Supplementary Video 1). In a frontal view, we were able to show how the fibers reached the pial surface at both sides of the floor plate before they bend caudally and criss-cross the floor plate (Figures 6C,D and Supplementary Video 1). In the *Wnt1^–/–^* mutant embryo, in a lateral view we confirmed the reduction in dopaminergic neurons and the thicker mHb fr that displayed an abnormal final trajectory (Figures 6E,F and Supplementary Video 2). Consequently, in a frontal view, we clearly detected the aberrant trajectory that the axons followed once they reached the isthmic territory and the abnormal floor plate cross of the fibers (Figures 6G,H and Supplementary Video 2).

### Altered Territories in the *Wnt1^–/–^* fr Pathway

**FIGURE 7:**
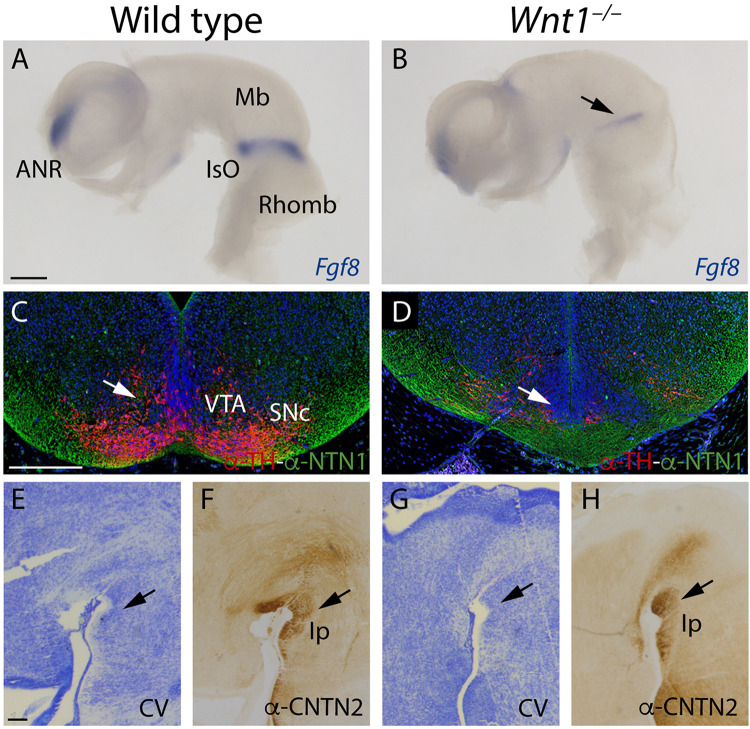
Study of the altered territories in the *Wnt1^–/–^* fr pathway. **(A,B)** *Fgf8 in situ* hybridization. Arrow in **(B)** shows a reduction of *Fgf8* expression in the mutant. **(C,D)** Immunofluorescence against TH and NTN1 in coronal sections of E15.5 brains. Arrows in **(C,D)** indicates the alterations observed in the mutant (NTN1 and TH^+^ cells reduction). **(E–H)** Sagittal sections of E15.5 brains, **(E,G)** Cresyl Violet staining, and **(F,H)** immunohistochemistry against CNTN2 (arrows in **F,H** indicate the interpeduncular nucleus). Three samples of each genotype have been analyzed. ANR, anterior neural ridge; Mb, midbrain; IsO, isthmic organizer; Rhomb, rhombencephalon; VTA, ventral tegmental area; SNc, substantia nigra pars compacta; Ip, interpeduncular nucleus; CV, Cresyl Violet. Scale bar: 200 μm.

The specification of the mesencephalic and rostral rhombencephalon is controlled by the isthmic organizer (IsO). This secondary organizer expresses a combination of morphogens that include *Wnt1* and *Fgf8*, among others. The alteration of any of these genes produces a strong malfunction of this vital organizer. The fr misdirection in the *Wnt1^–/–^* could be due to alterations in the specification of the territories that it must navigate. In order to proof this, we first studied the *Fgf8* expression pattern. In the wt at E10.5, *Fgf8* was expressed in the isthmic constriction and in the anterior neural ridge (Figure 7A). In the *Wnt1^–/–^* mutant, the *Fgf8* expression is strongly reduced but it was not absent (arrow in Figure 7B). Rostrally, the IsO induces the specification of the dopaminergic populations. As recently demonstrated (Company et al., 2021), the *Ntn1* expression by these populations are necessary for the correct fr trajectory. In the wt, in a midbrain frontal section we observed the correlation between the dopaminergic populations and the NTN1 distribution (Figure 7C). In the *Wnt1^–/–^* mutant, we confirmed the dramatic reduction of dopaminergic neurons and how this alteration produced a NTN1 deprivation in the territory (arrow in Figure 7D). Finally, we studied the Ip nucleus, final target of the mHb axons. We detected the Ip location by cresyl violet staining (arrow in Figure 7E) and the mHb axons terminals labeled by CNTN2 allowed us to confirm the Ip identification (arrow in Figure 7F). In the *Wnt1^–/–^* mutant embryo, the Ip area did not show any apparent alteration (arrow in Figure 7G). However, the CNTN2 labeling confirmed us the Ip localization (arrow in Figure 7H). The Ip nucleus was indeed specified but its neuronal distribution was slightly abnormal.

## Discussion

The Hb localization in the most dorsal aspect of the thalamic prosomere (Martinez-Ferre and Martinez, 2012) leads us to hypothesize that this important neuronal population must be under the control of the well-known dorsalizing morphogen activity produced by roof plate. *Wnt1*, among others, is one of these morphogenes (Navarro-Garberi et al., 2016; Brafman and Willert, 2017). Therefore, our aim in this work was to analyze the role of *Wnt1* in the development of the habenula and its efferent tract.

### *Wnt1* Effect in Habenular Development

Morphogenes are crucial for the proper development of neuronal populations at least in two aspects. On one hand, they specify the genetic cascades that direct the specific differentiation programs of the neuronal populations (Brafman and Willert, 2017). On the other hand, they regulate the proliferation rate of the neuroblast that will give rise to the different neurons (Cayuso and Martí, 2005).

Our results indicate that *Wnt1* is not directly related with the specification of the habenular neurons as we were able to identify all the different subnuclei that compose this complex. This result is in contrast to the differentiation changes observed in the dorsal Hb (equivalent to mammal mHb) of Zebrafish in the lack of *Wnt1* or the absence of ventral Hb (equivalent to mammal lHb) and mis-specification of the dorsal Hb in the complete blockage of the *Wnt* signaling in the *wls* mutant (Beretta et al., 2013; Hüsken and Carl, 2013; Kuan et al., 2015; Guglielmi et al., 2020). The strong differences in habenular organization and also the strong asymmetry found in Zebrafish may explain the phenotype variances found between these two vertebrate models. The alterations observed in the zebrafish wls mutant suggest that other Wnt members must be involved in the specification of the habenular territory. Nevertheless, the increased habenular volume in the mutant indicated that *Wnt1* is involved in the control of the number of habenular neurons. The phenotype observed can be due to two different phenomena. The *Wnt1* lack of function can induce a change in the differentiation program of the thalamic neuroblast and modify their destiny into habenular neurons (Martinez-Ferre et al., 2013). Another possibility is that this absence can modify the proliferation rate of the habenular progenitors and give rise by increment to a larger habenular complex (Cayuso and Martí, 2005).

Nevertheless, the fact that the mHb subpopulations did not change their size proportions among them prompted us to discard this first hypothesis. However, the strong increment in the proliferation rate of the habenular neuroblasts that we have shown supports the second hypothesis. The decrease in the thalamic territory could also be explained by a reduction in its proliferation rather than a change in their identity. Therefore, *Wnt1* is needed to determine the proliferation rate of the habenular precursors. It must be highlighted that the strongest effect was found in the mHb. The fact that the highest proliferation increase in the mutant was found at E12.5, time window when the mHb is generated (Funato et al., 2000; Belle et al., 2014). It is also striking that “single cell” RNAseq experiments demonstrated that the mHb neurons express several Lef/Tcf downstream genes while only *Tcf7l1*, a repressor of *Wnt1* target genes (Mao and Byers, 2011), is expressed in both mHb and lHb. Therefore, it is not surprising that the *Wnt1* absence has a deeper effect in the mHb compared with the lHb (Hashikawa et al., 2020; Wallace et al., 2020).

### *Wnt1* Effect on the fr

The defects found in the fr trajectory in the *Wnt1^–/–^* mouse display two different aspects. First, we detected a clear increment in the size of the tract and a slight disorganization of the fibers inside the fascicle. Obviously, the increment in size is due to the increased number of habenular neurons in the mutant. The lack of *Wnt1* did not produce changes in the differentiation of the habenular subnuclei but the increment in size and surface extension may account for the slight disorganization that we have observed in the sheath of the mutant fascicle. Second, we found a clear misdirection in the final fr trajectory. This phenotype can also be due to alterations in the surface molecules of the fr axons that alter their response to the surrounding navigation signals or to alterations in the specification of the territories that the axons must cross and therefore of the navigating signals (Company et al., 2021). The fact that all the different Hb subnuclei are specified suggest that the Hb axons contain the correct combination of receptors. However, it is well known that *Wnt1* lack of function produces severe alterations in the specification of the IsO (McMahon et al., 1992). As we have shown, the mutant IsO displayed a strong reduction of *Fgf8* expression, its main morphogen. Increasing reductions of FGF8 amount in the IsO resulted in escalating severe phenotypes at both sides of the organizer (Garda et al., 2001; Chi et al., 2003; Basson et al., 2008). Thus, a malfunction of this organizer produces strong differentiation impairments in the midbrain and rostral hindbrain. The effect on the surrounding territory is not symmetrical, the midbrain is more sensible than the hindbrain (Basson et al., 2008). In fact, the SNc and the VTA are strongly reduced in our non-functional IsO, due to the *Fgf8* reduction and *Wnt1* loss (Prakash et al., 2006; Smidt and Burbach, 2007; dos Santos and Smidt, 2011), meanwhile the Ip generated in rhombomere 1 is almost not affected (Basson et al., 2008). Recently, it has been proved that SNc and VTA are involved, via *Netrin1*, in the correct navigation of the fr axons through this territory (Company et al., 2021). The absence of a *Netrin1* correct signal must be responsible for the misdirection of a part of the fr axons. Some of them are still able to reach the Ip probably due to the fact that not all the dopaminergic neurons are lost (Wurst and Prakash, 2014). Thus, the *Wnt1* lack of function altered the IsO functionality which produced a strong reduction in SNc and VTA neurons. The miss-specification of this intermediate target of the fr axons must be responsible for their disrupted trajectory in our mutant model.

In summary, we can conclude that *Wnt1* is not directly related in the specification of the habenular neurons but it is responsible for the proliferation rate regulation of the habenular neuroblasts. Other morphogenes expressed in the diencephalic roof plate (like *BMPs* or other *Wnt* family members, must account for the determination of this neuronal population. This is supported by the results obtained with the *wls* mutant (protein involved in the WNT secretion) in zebrafish. The *wls* habenular phenotype is dramatic compared with the *Wnt1* mutant indicating that other *Wnt* members must be involved in the Hb specification (Kuan et al., 2015). The *Wnt1* lack of function produced and altered IsO, this alteration produced a miss differentiation of the territory crossed by the fr resulting in an erroneous direction of the fascicle.

## Data Availability Statement

The original contributions presented in the study are included in the article/Supplementary Material, further inquiries can be directed to the corresponding author/s.

## Ethics Statement

The animal study was reviewed and approved by Universidad Miguel Hernández OIR Committee (2016/VSC/PEA/00190).

## Author Contributions

SM, DE, and EP conceived, obtained funding, and designed the experiments. VC, AM-C, AA-C, RM-R, and FA-G performed the experiments. DE and EP analyzed the data. EP wrote the article. All authors had full access to all the data in the study and take responsibility for the integrity of the data and the accuracy of the data analysis.

## Conflict of Interest

The authors declare that the research was conducted in the absence of any commercial or financial relationships that could be construed as a potential conflict of interest.

## Publisher’s Note

All claims expressed in this article are solely those of the authors and do not necessarily represent those of their affiliated organizations, or those of the publisher, the editors and the reviewers. Any product that may be evaluated in this article, or claim that may be made by its manufacturer, is not guaranteed or endorsed by the publisher.

## References

[B1] Agetsuma M. Aizawa H. Aoki T. Nakayama R. Takahoko M. Goto M. (2010). The habenula is crucial for experience-dependent modification of fear responses in zebrafish. *Nat. Neurosci.* 13 1354–1356. 10.1038/nn.2654 20935642

[B2] Aizawa H. Amo R. Okamoto H. (2011). Phylogeny and ontogeny of the habenular structure. *Front. Neurosci.* 5:138. 10.3389/fnins.2011.00138 22203792 PMC3244072

[B3] Amo R. Fredes F. Kinoshita M. Aoki R. Aizawa H. Agetsuma M. (2014). The habenulo-raphe serotonergic circuit encodes an aversive expectation value essential for adaptive active avoidance of danger. *Neuron* 84 1034–1048. 10.1016/J.NEURON.2014.10.035 25467985

[B4] Andres K. Von Düring M. Veh R. (1999). Subnuclear organization of the rathabenular complexes. *J. Comp. Neurol.* 407 130–150. 10.1002/(sici)1096-9861(19990428)407:1<130::aid-cne10<3.0.co;2-8 10213193

[B5] Barth K. A. Kishimoto Y. Rohr K. B. Seydler C. Schulte-Merker S. Wilson S. W. (1999). Bmp activity establishes a gradient of positional information throughout the entire neural plate. *Development* 126 4977–4987. 10529416 10.1242/dev.126.22.4977

[B6] Basson M. A. Echevarria D. Ahn C. P. Sudarov A. Joyner A. L. Mason I. J. (2008). Specific regions within the embryonic midbrain and cerebellum require different levels of FGF signaling during development. *Development* 135 889–898. 10.1242/dev.011569 18216176 PMC2555978

[B7] Batalla A. Homberg J. R. Lipina T. V. Sescousse G. Luijten M. Ivanova S. A. (2017). The role of the habenula in the transition from reward to misery in substance use and mood disorders. *Neurosci. Biobehav. Rev.* 80 276–285. 10.1016/j.neubiorev.2017.03.019 28576510

[B8] Belle M. Godefroy D. Dominici C. Heitz-Marchaland C. Zelina P. Hellal F. (2014). A simple method for 3D analysis of immunolabeled axonal tracts in a transparent nervous system. *Cell Rep.* 9 1191–1201. 10.1016/j.celrep.2014.10.037 25456121

[B9] Benarroch E. E. (2015). Habenula. *Neurology* 85 992–1000. 10.1212/WNL.0000000000001937 26291286

[B10] Bengoa-Vergniory N. Kypta R. M. (2015). Canonical and noncanonical Wnt signaling in neural stem/progenitor cells. *Cell. Mol. Life Sci.* 72 4157–4172. 10.1007/s00018-015-2028-6 26306936 PMC11113751

[B11] Beretta C. A. Dross N. Bankhead P. Carl M. (2013). The ventral habenulae of zebrafish develop in prosomere 2 dependent on Tcf7l2 function. *Neural Dev.* 8:19. 10.1186/1749-8104-8-19 24067090 PMC3827927

[B12] Bianco I. Wilson S. (2009). The habenular nuclei: a conserved asymmetric relay station in the vertebrate brain. *Philos. Trans. R. Soc. Lond. B. Biol. Sci.* 364, 1005–1020. 10.1098/RSTB.2008.0213 19064356 PMC2666075

[B13] Brafman D. Willert K. (2017). Wnt/β-catenin signaling during early vertebrate neural development. *Dev. Neurobiol.* 77 1239–1259. 10.1002/dneu.22517 28799266 PMC5819875

[B14] Cayuso J. Martí E. (2005). Morphogens in motion: growth control of the neural tube. *J. Neurobiol.* 64 376–387. 10.1002/NEU.20169 16041754

[B15] Cherng B.-W. Islam T. Torigoe M. Tsuboi T. Okamoto H. (2020). The dorsal lateral habenula-interpeduncular nucleus pathway is essential for left-right-dependent decision making in zebrafish. *Cell Rep.* 32:108143. 10.1016/J.CELREP.2020.108143 32937118

[B16] Chi C. L. Martinez S. Wurst W. Martin G. R. (2003). The isthmic organizer signal FGF8 is required for cell survival in the prospective midbrain and cerebellum. *Development* 130 2633–2644. 10.1242/dev.00487 12736208

[B17] Ciani L. Salinas P. C. (2005). WNTs in the vertebrate nervous system: from patterning to neuronal connectivity. *Nat. Rev. Neurosci.* 6 351–362. 10.1038/nrn1665 15832199

[B18] Company V. Andreu-Cervera A. Madrigal M. P. Andrés B. Almagro-García F. Chédotal A. (2021). Netrin 1-mediated role of the substantia nigra pars compacta and ventral tegmental area in the guidance of the medial habenular axons. *Front. Cell Dev. Biol.* 9:1183. 10.3389/fcell.2021.682067 34169076 PMC8217627

[B19] Contestabile R. A. Flumerfelt B. A. (1981). Afferent connections of the interpeduncular nucleus and the topographic organization of the habenulo-interpeduncular pathway: an HRP study in the rat. *J. Comp. Neurol.* 196 253–270. 10.1002/cne.901960206 7217357

[B20] dos Santos M. T. A. Smidt M. P. (2011). En1 and Wnt signaling in midbrain dopaminergic neuronal development. *Neural Dev.* 6:21. 10.1186/1749-8104-6-23 21569278 PMC3104484

[B21] Fakhoury M. (2018). The dorsal diencephalic conduction system in reward processing: spotlight on the anatomy and functions of the habenular complex. *Behav. Brain Res.* 348 115–126. 10.1016/j.bbr.2018.04.018 29684476

[B22] Fore S. Palumbo F. Pelgrims R. Yaksi E. (2018). Information processing in the vertebrate habenula. *Semin. Cell Dev. Biol.* 78, 130–139. 10.1016/j.semcdb.2017.08.019 28797836

[B23] Funato H. Saito-Nakazato Y. Takahashi H. (2000). Axonal growth from the habenular nucleus along the neuromere boundary region of the diencephalon is regulated semaphorin 3F and netrin-1. *Mol. Cell. Neurosci.* 16 206–220. 10.1006/mcne.2000.0870 10995548

[B24] Garda A. L. Echevarría D. Martínez S. (2001). Neuroepithelial co-expression of Gbx2 and Otx2 precedes Fgf8 expression in the isthmic organizer. *Mech. Dev.* 101 111–118. 10.1016/S0925-4773(00)00567-0 11231064

[B25] Grillner S. von Twickel A. Robertson B. (2018). The blueprint of the vertebrate forebrain – with special reference to the habenulae. *Semin. Cell Dev. Biol.* 78, 103–106. 10.1016/j.semcdb.2017.10.023 29107476

[B26] Guglielmi L. BüHler A. Moro E. Argenton F. Poggi L. Carl M. (2020). Temporal control of Wnt signaling is required for habenular neuron diversity and brain asymmetry. *Development* 147:dev182865. 10.1242/dev.182865 32179574

[B27] Hashikawa Y. Hashikawa K. Rossi M. A. Basiri M. L. Liu Y. Johnston N. L. (2020). Transcriptional and spatial resolution of cell types in the mammalian habenula. *Neuron* 106 743–758.e5. 10.1016/j.neuron.2020.03.011 32272058 PMC7285796

[B28] Hikosaka O. (2010). The habenula: from stress evasion to value-based decision-making. *Nat. Rev. Neurosci.* 11 503–513. 10.1038/nrn2866 20559337 PMC3447364

[B29] Hikosaka O. Sesack S. R. Lecourtier L. Shepard P. D. (2008). Habenula: crossroad between the basal ganglia and the limbic system. *J. Neurosci.* 28, 11825–11829. 10.1523/JNEUROSCI.3463-08.2008 19005047 PMC2613689

[B30] Hu H. Cui Y. Yang Y. (2020). Circuits and functions of the lateral habenula in health and in disease. *Nat. Rev. Neurosci.* 21 277–295. 10.1038/s41583-020-0292-4 32269316

[B31] Hüsken U. Carl M. (2013). The Wnt/beta-catenin signaling pathway establishes neuroanatomical asymmetries and their laterality. *Mech. Dev.* 130 330–335. 10.1016/J.MOD.2012.09.002 23022991

[B32] Jesuthasan S. (2012). Fear, anxiety, and control in the zebrafish. *Dev. Neurobiol.* 72 395–403. 10.1002/DNEU.20873 22328274

[B33] Jhou T. C. Geisler S. Marinelli M. Degarmo B. A. Zahm D. S. (2009). The mesopontine rostromedial tegmental nucleus: a structure targeted by the lateral habenula that projects to the ventral tegmental area of Tsai and substantia nigra compacta. *J. Comp. Neurol.* 513 566–596. 10.1002/cne.21891 19235216 PMC3116663

[B34] Kazanskaya O. Glinka A. Niehrs C. (2000). The role of Xenopus dickkopf1 in prechordal plate specification and neural patterning. *Development* 127 4981–4992. 10.1242/DEV.127.22.4981 11044411

[B35] Klemm W. R. (2004). Habenular and interpeduncularis nuclei: shared components in multiple-function networks. *Med. Sci. Monit.* 10 261–274. 15507867

[B36] Koppensteiner P. Galvin C. Ninan I. (2016). Development- and experience-dependent plasticity in the dorsomedial habenula. *Mol. Cell. Neurosci.* 77 105–112. 10.1016/j.mcn.2016.10.006 27793697 PMC5124526

[B37] Kuan Y. S. Roberson S. Akitake C. M. Fortuno L. Gamse J. Moens C. (2015). Distinct requirements for Wntless in habenular development. *Dev. Biol.* 406 117–128. 10.1016/J.YDBIO.2015.06.006 26116173 PMC4639407

[B38] Lee A. Mathuru A. S. Teh C. Kibat C. Korzh V. Penney T. B. (2010). The habenula prevents helpless behavior in larval zebrafish. *Curr. Biol.* 20 2211–2216. 10.1016/J.CUB.2010.11.025 21145744

[B39] Loonen A. J. M. Ivanova S. A. (2019). Evolution of circuits regulating pleasure and happiness with the habenula in control. *CNS Spectr.* 24 233–238. 10.1017/S1092852917000748 29091022

[B40] Mao C. D. Byers S. W. (2011). Cell-context dependent TCF/LEF expression and function: alternative tales of repression, de-repression and activation potentials. *Crit. Rev. Eukaryot. Gene Expr.* 21:207. 10.1615/critreveukargeneexpr.v21.i3.10 PMC3434703 22111711

[B41] Martinez-Ferre A. Martinez S. (2012). Molecular regionalization of the diencephalon. *Front. Neurosci.* 6:73. 10.3389/fnins.2012.00073 22654731 PMC3360461

[B42] Martinez-Ferre A. Navarro-Garberi M. Bueno C. Martinez S. (2013). Wnt signal specifies the intrathalamic limit and its organizer properties by regulating Shh induction in the alar plate. *J. Neurosci.* 33, 3967–3980. 10.1523/JNEUROSCI.0726-12.2013 23447606 PMC6619321

[B43] Masai I. Heisenberg C. P. Barth K. A. Macdonald R. Adamek S. Wilson S. W. (1997). Floating head and masterblind regulate neuronal patterning in the roof of the forebrain. *Neuron* 18 43–57. 10.1016/S0896-6273(01)80045-3 9010204

[B44] Mathuru A. S. (2018). A little rein on addiction. *Semin. Cell Dev. Biol.* 78 120–129. 10.1016/j.semcdb.2017.09.030 28986065

[B45] Mathuru A. S. Jesuthasan S. (2013). The medial habenula as a regulator of anxiety in adult zebrafish. *Front. Neural Circuits* 7:99. 10.3389/FNCIR.2013.00099 23750127 PMC3664309

[B46] McMahon A. P. Joyner A. L. Bradley A. McMahon J. A. (1992). The midbrain-hindbrain phenotype of Wnt-1- Wnt-1- mice results from stepwise deletion of engrailed-expressing cells by 9.5 days postcoitum. *Cell* 69 581–595. 10.1016/0092-8674(92)90222-X 1534034

[B47] Moreno-Bravo J. A. Martinez-Lopez J. E. Madrigal M. P. Kim M. Mastick G. S. Lopez-Bendito G. (2016). Developmental guidance of the retroflex tract at its bending point involves Robo1-Slit2-mediated floor plate repulsion. *Brain Struct. Funct.* 221 665–678. 10.1007/s00429-014-0932-4 25366972 PMC4485949

[B68] Murcia-Ramón R. Company V. Juárez-Leal I. Andreu-Cervera A. Almagro-García F. Martínez S. (2020). Neuronal tangential migration from *Nkx2.1*-positive hypothalamus. *Brain Struct. Funct*. 225, 2857–2869. 10.1007/s00429-020-02163-x 33145610 PMC7674375

[B48] Nakajima M. Mori H. Nishikawa C. Tsuruta M. Okuyama S. Furukawa Y. (2013). Psychiatric disorder-related abnormal behavior and habenulointerpeduncular pathway defects in Wnt1-cre and Wnt1-GAL4 double transgenic mice. *J. Neurochem.* 124 241–249. 10.1111/jnc.12085 23134367

[B49] Nakajo H. Tsuboi T. Okamoto H. (2020). The behavioral paradigm to induce repeated social defeats in zebrafish. *Neurosci. Res.* 161 24–32. 10.1016/J.NEURES.2019.11.004 31711781

[B50] Navarro-Garberi M. Bueno C. Martinez S. (2016). Wnt1 signal determines the patterning of the diencephalic dorso-ventral axis. *Brain Struct. Funct.* 221 3693–3708. 10.1007/s00429-015-1126-4 26452989

[B51] Okamoto H. Agetsuma M. Aizawa H. (2012). Genetic dissection of the zebrafish habenula, a possible switching board for selection of behavioral strategy to cope with fear and anxiety. *Dev. Neurobiol.* 72 386–394. 10.1002/DNEU.20913 21567982

[B52] Okamoto H. Cherng B. W. Nakajo H. Chou M. Y. Kinoshita M. (2021). Habenula as the experience-dependent controlling switchboard of behavior and attention in social conflict and learning. *Curr. Opin. Neurobiol.* 68 36–43. 10.1016/j.conb.2020.12.005 33421772

[B53] Pandey S. Shekhar K. Regev A. Schier A. F. (2018). Comprehensive identification and spatial mapping of habenular neuronal types using single-Cell RNA-Seq. *Curr. Biol.* 28 1052–1065.e7. 10.1016/J.CUB.2018.02.040 29576475 PMC6042852

[B54] Prakash N. Brodski C. Naserke T. Puelles E. Gogoi R. Hall A. (2006). A Wnt1-regulated genetic network controls the identity and fate of midbrain-dopaminergic progenitors in vivo. *Development* 133 89–98. 10.1242/dev.02181 16339193

[B55] Puelles L. (2019). Survey of midbrain, diencephalon, and hypothalamus neuroanatomic terms whose prosomeric definition conflicts with columnar tradition. *Front. Neuroanat.* 13:20. 10.3389/FNANA.2019.00020 30873012 PMC6402269

[B56] Ramón y Cajal S. (1909). *Histologie du Système Nerveux de L’homme & des Vertébrés*. Paris: Maloine. 10.5962/bhl.title.48637.

[B57] Regan J. C. Concha M. L. Roussigne M. Russell C. Wilson S. W. (2009). An Fgf8-dependent bistable cell migratory event establishes CNS asymmetry. *Neuron* 61 27–34. 10.1016/j.neuron.2008.11.030 19146810 PMC2790412

[B58] Roman E. Weininger J. Lim B. Roman M. Barry D. Tierney P. (2020). Untangling the dorsal diencephalic conduction system: a review of structure and function of the stria medullaris, habenula and fasciculus retroflexus. *Brain Struct. Funct.* 225 1437–1458. 10.1007/s00429-020-02069-8 32367265

[B59] Schmidt E. R. E. Pasterkamp R. J. (2017). The molecular mechanisms controlling morphogenesis and wiring of the habenula. *Pharmacol. Biochem. Behav.* 162 29–37. 10.1016/j.pbb.2017.08.008 28843424

[B60] Schmidt E. R. E. Brignani S. Adolfs Y. Lemstra S. Demmers J. Vidaki M. (2014). Subdomain-mediated axon-axon signaling and chemoattraction cooperate to regulate afferent innervation of the lateral habenula. *Neuron* 83 372–387. 10.1016/j.neuron.2014.05.036 25033181

[B61] Smidt M. P. Burbach J. P. H. (2007). How to make a mesodiencephalic dopaminergic neuron. *Nat. Rev. Neurosci.* 8 21–32. 10.1038/nrn2039 17180160

[B62] Sutherland R. J. (1982). The dorsal diencephalic conduction system: a review of the anatomy and functions of the habenular complex. *Neurosci. Biobehav. Rev.* 6 1–13. 10.1016/0149-7634(82)90003-3 7041014

[B63] Taciak B. Pruszynska I. Kiraga L. Bialasek M. Krol M. (2018). Wnt signaling pathway in development and cancer. *J. Physiol. Pharmacol.* 69 185–196. 10.26402/jpp.2018.2.07 29980141

[B64] Wagner F. French L. Veh R. W. (2016). Transcriptomic-anatomic analysis of the mouse habenula uncovers a high molecular heterogeneity among neurons in the lateral complex, while gene expression in the medial complex largely obeys subnuclear boundaries. *Brain Struct. Funct.* 221 39–58. 10.1007/s00429-014-0891-9 25244943

[B65] Wallace M. L. Huang K. W. Hochbaum D. Hyun M. Radeljic G. Sabatini B. L. (2020). Anatomical and single-cell transcriptional profiling of the murine habenular complex. *Elife* 9:e51271. 10.7554/eLife.51271 32043968 PMC7012610

[B66] Wurst W. Prakash N. (2014). Wnt1-regulated genetic networks in midbrain dopaminergic neuron development. *J. Mol. Cell Biol.* 6 34–41. 10.1093/JMCB/MJT046 24326514

[B67] Zahm D. S. Root D. H. (2017). Review of the cytology and connections of the lateral habenula, an avatar of adaptive behaving. *Pharmacol. Biochem. Behav.* 162 3–21. 10.1016/j.pbb.2017.06.004 28647565 PMC5659881

